# Clinical Evaluation of a Multi-Omic Diagnostic Model for Early-Stage Ovarian Cancer Detection

**DOI:** 10.3390/diagnostics15172225

**Published:** 2025-09-02

**Authors:** Robert A. Law, Brendan M. Giles, Rachel Culp-Hill, Enkhtuya Radnaa, Mattie Goldberg, Charles M. Nichols, Maria Wong, Connor Hansen, Collin Hill, Katrin Eurich, Emily Prendergast, Kian Behbakht, Benjamin G. Bitler, Anna Jeter, Vuna S. Fa, James Robert White, Kevin Elias, Abigail McElhinny

**Affiliations:** 1 AOA Dx, Denver, CO 80221, USA; robert.law@aoadx.com (R.A.L.); brendan.giles@aoadx.com (B.M.G.); rachel.hill@aoadx.com (R.C.-H.); enkhtuya.radnaa@aoadx.com (E.R.); mattie.goldberg@aoadx.com (M.G.); charles.nichols@aoadx.com (C.M.N.); maria.wong@aoadx.com (M.W.); connor.hansen@aoadx.com (C.H.); collin.hill@aoadx.com (C.H.); anna.jeter@aoadx.com (A.J.); vuna.fa@aoadx.com (V.S.F.); 2 Gynecologic Oncology, UT Health San Antonio, San Antonio, TX 78229, USA; katrineurich@gmail.com; 3 Gynecologic Oncology, Intermountain Health Intermountain Medical Center, Salt Lake City, UT 84111, USA; emily.prendergast@imail.org; 4 Department of Obstetrics and Gynecology, Division of Reproductive Sciences, University of Colorado Anschutz Medical Campus, Denver, CO 80217, USA; kian.behbakht@cuanschutz.edu (K.B.); benjamin.bitler@cuanschutz.edu (B.G.B.); 5 Resphera Biosciences, Baltimore, MD 21231, USA; jwhite@respherabio.com; 6 Obstetrics & Gynecology Institute, Department of Biomedical Engineering at the Lerner Research Institute, Cleveland Clinic, Cleveland, OH 44195, USA; eliask2@ccf.org

**Keywords:** ovarian cancer, early detection, diagnostic biomarkers, multi-omics, lipidomics, machine learning, mass spectrometry

## Abstract

**Background/Objectives**: Ovarian cancer (OC) is frequently diagnosed at an advanced stage due to the nonspecific nature of its symptoms. While population-wide screening has failed to reduce mortality, timely diagnosis in symptomatic women remains a promising and underutilized strategy to improve clinical outcomes. The aim of this study was to develop a sensitive, scalable biomarker assay to improve early-stage detection in symptomatic women. **Methods**: A multi-omic diagnostic model was developed using serum samples from symptomatic women. Lipidomic profiles were generated by liquid chromatography–mass spectrometry (LC-MS), and protein levels were measured using immunoassays. Statistical and machine learning approaches were applied to assess diagnostic performance across disease stages and subtypes. **Results**: The multi-omic model demonstrated robust performance across a clinically challenging population, with both lipid and protein data necessary for detecting OC across a range of stages and subtypes. The model achieved 98.7% sensitivity in early-stage OC and 98.6% across a range of OC subtypes and stages at 70% fixed specificity, which represented significant improvements over CA125 in the same cohort. In addition, in a small subset of samples, lipid and protein profiles from OC patients undergoing treatment differed from untreated patients and controls, suggesting that this approach may also be useful in other aspects of clinical management, such as treatment monitoring. **Conclusions**: This multi-omic assay offers a promising solution to accelerate diagnosis, improve early detection, and potentially reduce OC mortality.

## 1. Introduction

Ovarian cancer (OC) has the highest case fatality rate among common gynecologic cancers and remains a major contributor to female cancer mortality worldwide [1,2]. This high fatality rate is primarily driven by late-stage diagnosis; over 70% of cases are detected only after the disease has spread beyond the pelvis [3,4]. Despite longstanding recognition of this diagnostic challenge, no validated early detection tools exist for average-risk women [5]. OC patients often experience subtle abdominal symptoms, such as bloating, pelvic discomfort, or early satiety, appearing 3–36 months prior to diagnosis [6,7,8,9]. These symptoms overlap with those of various benign genitourinary (GU) and gastrointestinal (GI) disorders. In a survey of 1700 women diagnosed with OC, most reported receiving prior diagnoses such as irritable bowel syndrome, constipation, urinary tract infections, stress, or depression [6], leading to delays in investigation and prolonged diagnostic intervals [10]. The nonspecific nature of OC symptoms, combined with its low incidence, further complicates early detection [11].

In current clinical practice in the U.S., women presenting with these symptoms typically undergo a fragmented sequence of evaluations across multiple specialties, such as primary care, gynecology, and gastroenterology, before imaging or biomarker testing for OC is initiated [9,12,13]. This process results in an average diagnostic interval of approximately nine months [14,15]. During this diagnostic interval, high-grade serous carcinoma (HGSC), the most common and aggressive subtype, doubles in volume every 2.2 to 4 months [16,17]. These factors contribute to the predominance of late-stage diagnoses and higher mortality rates. Notably, OC cases detected at early stages have five-year survival rates exceeding 90% [18], but existing biomarkers such as CA125 and HE4 are cleared for monitoring applications only and demonstrate limited sensitivity for early-stage disease: Blood tests for CA125 detect only 50–60% of stage I/II cases, and HE4 performance varies based on menopausal status and presence of comorbidities [19,20]. Additionally, elevated CA125 levels can occur in common benign conditions such as uterine fibroids, pelvic inflammatory disease, endometriosis, and adenomyosis [21]. These limitations emphasize the urgent clinical need for more sensitive, specific, and accessible front-line diagnostics to guide timely and accurate clinical decision-making at symptom presentation.

Previous studies have demonstrated the ability to distinguish cancer from non-cancer samples via immunoassays using tumor-associated lipids GD2 and GD3 gangliosides in symptomatic women [22]. Building on these findings here, the approach was broadened to include a wider range of lipid species and to adopt a multi-omic strategy that integrates complementary molecular classes to improve early detection. Multi-omic approaches that combine orthogonal molecular signals can enhance diagnostic performance by capturing distinct biological processes that may be overlooked when analyzing individual biomarker classes alone [23].

To test this hypothesis, an untargeted lipidomics profiling method was developed using liquid chromatography–mass spectrometry (LC-MS), capable of surveying hundreds of lipid species in minimal serum volumes (<20 µL). This broad-spectrum approach was applied to a large, clinically representative cohort (*N* = 509) composed of early- and late-stage OC, benign adnexal masses, other gynecologic/gastrointestinal disorders, and age-representative controls from female donors who are otherwise “healthy”. To contextualize multi-omic biomarker performance against the current standard of care, CA125 was also measured via immunoassay and cut-offs were applied per current guidelines.

To assess whether combining protein and lipid markers interpreted through advanced algorithms could improve diagnostic accuracy for early-stage OC, particularly in women presenting with vague abdominal symptoms (VAS), a serum-based assay was developed that integrates lipid and protein biomarkers within a machine learning (ML) platform. This multi-omic approach aims to enhance early-stage detection sensitivity with high specificity, beyond the capabilities of current methods, thereby enabling triage of women presenting with VAS and expedite their clinical assessment. The assay’s performance was evaluated in a large, clinically diverse cohort representative of real-world differential diagnoses encountered during OC workups.

The results of this study show that the lipid–protein ML model significantly outperforms standard-of-care biomarkers in distinguishing early-stage OC within the spectrum of non-OC controls representing the symptomatic population. This supports its potential as a front-line diagnostic tool to ensure women presenting with signs and symptoms of OC receive appropriate care in a timely manner. Collectively, the results represent a significant advancement toward addressing the critical unmet need for early, accurate, and accessible OC detection at the time of symptom presentation, thereby potentially improving patient outcomes.

## 2. Materials and Methods

### 2.1. Study Design/Human Specimens

To explore lipidomic biomarkers indicative of OC across its clinical spectrum, a biomarker discovery cohort was assembled to include serum specimens from patients representing various OC stages and histological subtypes (Table 1). This cohort included both early- and late-stage disease to capture lipid species common to different OC presentations. Notably, over 90% of OC samples were collected prior to any cancer-directed therapy, reflecting the typical symptomatic patient population encountered in clinical settings.

In addition to OC, the cohort included individuals with benign gynecological conditions that often present with symptoms overlapping those of OC, including endometriosis, uterine fibroids, and both simple and complex adnexal masses (Table S1). To further account for clinical heterogeneity, gastrointestinal (GI) disorders such as ulcerative colitis and Crohn’s disease were also represented (Table S2). Age-matched healthy female donors were incorporated to establish normative baseline levels for lipid and protein biomarkers. Cohort demographics including age distribution and race/ethnicity are summarized in Table 2. Although classified as “healthy,” most donors had common clinical diagnoses such as hypertension and arthritis, reflective of the general population. Detailed age distribution of the cohort stratified by subgroup is provided in Table S3.

Serum specimens were sourced primarily from the University of Colorado Gynecologic Tissue and Fluid Bank (IRB #07-935, 21-4787) and supplemented with samples from commercial biobanks (iSpecimen, Woburn, MA, USA and Aspira, Austin, TX, USA). Key clinical and demographic data, including diagnosis, stage, histological subtype, age, sex, ethnicity, smoking status, and comorbidities, were available for most participants. However, information regarding blood processing protocols, fasting status, body mass index, and use of medications that could affect lipid metabolism was largely unavailable. All specimens were obtained and utilized after being de-identified by the vendor or academic collaborator. No PHI was available or shared.

The use of multiple specimen sources early in assay development provides opportunities to assess potential molecular or analytical variability arising from site-specific collection or pre-analytical differences, thereby enhancing the robustness of biomarker performance evaluation. To minimize bias during assay processing and model development, sample identifiers and group labels (e.g., controls vs. OC) were blinded to both laboratory personnel and data analysts until all measurements and preliminary modeling were complete.

### 2.2. Sample Extraction

For lipidomic analysis, serum samples were extracted using 100% LC-MS-grade methanol (Fisher Scientific, Pittsburgh, PA, USA). To facilitate quality control, methanol was spiked with synthetic lipid standards: 45 ng/mL C18:0-d7 GD1b Ceramide (d18:1/18:0-d7), 23 ng/mL C18:0-d7 GD3 Ceramide (d18:1/18:0-d7), and a 1:11 dilution of SPLASH™ LIPIDOMIX™ Mass Spec Standard, according to manufacturer’s recommendations (Avanti Research, Birmingham, AL, USA). For each extraction, 20 µL serum was transferred into a 2 mL Eppendorf tube. Briefly, 180 µL of room temperature spiked methanol was added to each tube. Samples were vortexed at maximum speed for 30 s and centrifuged at 18,000× *g* for 10 min at 4 °C. The resulting supernatant was transferred into autosampler vials equipped with 300 µL glass inserts. Extracted samples were stored at −20 °C until LC-MS analysis.

### 2.3. LC-MS Specifications

Commercial reagents were purchased from Fisher Scientific (Pittsburgh, PA, USA). Sample analysis was performed by ultra-high-pressure liquid chromatography–mass spectrometry (UHPLC-MS—Vanquish and Orbitrap Exploris 240, Thermo Fisher, San Jose, CA, USA and Bremen, Germany) using reversed-phase chromatography and electrospray ionization (ESI) in negative mode. Then, 15 µL sample extract was loaded onto a Kinetex 2.6 µm C18 100A LC Column (100 × 2.1 mm—Phenomenex, Torrance, CA, USA) at 40 °C. A 20 min gradient at 320 µL/min (40–90% B, A: 60:40 methanol/water + 10 mM ammonium formate, B: 90:10 isopropanol/methanol + 10 mM ammonium formate) was used to elute lipids of interest. Three separate mass spectrometry experiments were run within the gradient. For experiment #1, from 0 to 4 min the MS performed Full Scan analysis at a resolution of 180,000 with a scan range of 90–500 *m/z*. For experiment #2, from 0 to 4 min the MS performed Full Scan analysis at a resolution of 120,000 with a scan range of 500–1700 *m/z*. For experiment #3, from 4 to 20 min the MS performed Full Scan analysis at a resolution of 120,000 with a scan range of 500–1700 *m/z*. Additionally, ddMS2 was performed using 5 dependent scans with a resolution of 22,500 and a scan range from 90 to 900 *m/z*. HCD collision energy (%) was set to 20 with an isolation window of 2 *m/z*. EASY-IC^TM^ (Thermo Fisher, San Jose, CA, USA) was used for all Full Scan experiments. Calibration was performed prior to analysis using Pierce™ FlexMix™ Calibration Solution (Thermo Fisher, San Jose, CA, USA). A global sample-level monitoring technical pooled mixture was injected every 10 samples to assess the precision of the LC-MS during the run. Internal standards maintained a coefficient of variation (CV) < 10% throughout the run. The initial cohort included 519 samples, but 10 were excluded during analysis due to technical issues that disrupted data acquisition, such as insufficient sample volume, internal control failures, instrument errors, or corrupted files. These excluded samples comprised 2 healthy, 3 benign, 1 GI, 2 early-stage OC, and 2 late-stage OC cases.

### 2.4. LCMS Data Analysis

For ganglioside and fatty acid analysis, raw data files were converted to mzML files using MS Convert (ProteoWizard, v3.0). Metabolite assignments using isotopologue distributions and expected natural abundances of ^13^C isotopes were performed using MAVEN (v2.10.21, Princeton, NJ, USA). Ganglioside identifications were confirmed using ganglioside-specific fragment moieties. Data were also analyzed using LipidSearch (v5.1, Thermo Fisher Scientific, Waltham, MA, USA) to perform relative quantitation and metabolite assignment against publicly available lipid databases.

### 2.5. Protein Immunoassays

A panel of protein markers was selected and designed based on the literature. Commercial RUO immunoassay kits for cancer antigen 125 (CA125), human epididymis protein 4 (HE4), apolipoprotein A1 (ApoA1), beta-2 microglobulin (B2M), and folate receptor alpha (FOLR1) were obtained from R&D Biosystems (BioTechne, Minneapolis, MN, USA). The mucin 1 (MUC1) assay kit was sourced from Invitrogen (Thermo Fisher Scientific, Waltham, MA, USA). All kits were analytically verified prior to use, following manufacturer’s protocols. Protein immunoassays were performed on unextracted serum from each specimen. The total serum volume required to test the full panel of protein biomarkers was <500 µL per sample. Repeat assays were conducted when replicate variability exceeded a 20% coefficient of variation (CV).

For exploratory analysis of proteins comparing OC specimens that had undergone treatment compared with untreated specimens, protein abundance values for the six biomarkers were auto-scaled (z-score normalized) across the cohort. Group-level mean abundance values were then computed for four stage- and treatment-defined groups: early-stage untreated (Early OC), late-stage untreated (Late OC), early-stage treated (Early OC Treat), and late-stage treated (Late OC Treat). These values were visualized in a composite heatmap alongside lipid features to assess global molecular shifts associated with treatment status.

### 2.6. Statistical Analysis and Feature Selection

Features were excluded if they were present in < 10% of samples within the cohort, not assigned a formula or compound name, or the coefficient of variation exceeded 50% in all technical pooled mixtures. Serum analyte feature data were joined with subject-level metadata including disease category, disease subtype, stage, specimen source, age, and other available information. Univariate statistical analysis was performed for each feature including calculation of mean, median, standard deviation, and 95% CI, as well as comparisons between subject groups of interest utilizing the Mann–Whitney U test and one-way ANOVA, with further calculation of log2 fold change values and classification capacity by AUC. Statistical comparisons were performed for different variables including primary disease category and cancer stage. Machine learning model training was performed between primary groups of interest using 20-fold cross-validation with logistic regression. To minimize overfitting and assess generalizability, we reserved independent hold-out sets during model development, which were excluded from all training and feature selection steps. Model performance was subsequently determined on these hold-out samples. Features were ranked and selected based on their initial univariate performance measures and then log10-transformed prior to model training. Statistical significance was determined as follows: ns (*p* > 0.05), * (*p* ≤ 0.05), ** (*p* ≤ 0.01), *** (*p* ≤ 0.001), and **** (*p* ≤ 0.0001).

## 3. Results

### 3.1. CA125 Performance in Pre- and Postmenopausal Women

The cohort composition, detailed in Table 1, encompasses a broad spectrum of OC stages and subtypes, alongside diverse control groups that reflect the complexity of symptomatic women presenting with signs and symptoms of OC. To our knowledge, this represents the first cohort of its kind used for molecular profiling in this symptomatic population.

CA125 was used as a benchmark to confirm the overall cohort and specimen quality, with its performance consistent with published literature (Table 3).

Using the standard 35 U/mL cut-off, CA125 demonstrated moderate overall sensitivity, with 67.5% sensitivity for early-stage OC and 76.2% sensitivity across all OC cases irrespective of age. This disparity has important clinical implications, as premenopausal women exhibit lower rates of cancer but higher rates of symptoms and adnexal masses [26], complicating timely and accurate diagnosis.

CA125 is widely employed in clinical practice across various laboratories and kit/reagent configurations, resulting in real-world performance that often falls short of the controlled settings observed in clinical studies [27]. Variability in CA125 assay implementation and interpretation contributes to inconsistent sensitivity and specificity, with a notable reduction in specificity among patients with benign adnexal masses, consistent with prior reports. Our cohort mirrored these limitations, underscoring the shortcomings of CA125 as a standalone biomarker for OC detection.

### 3.2. Feature Space and Multi-Omic Modeling

**Figure 1:**
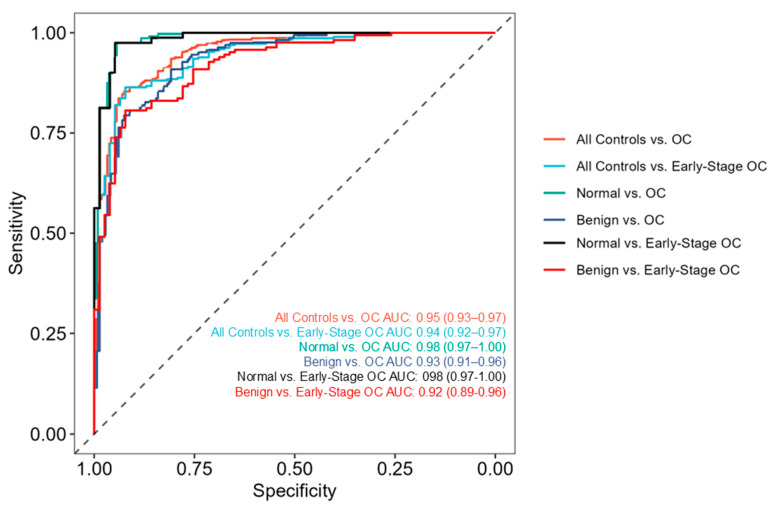
**ROC curves for various populations of interest.** AUCs (±95% CI) are shown above for the classification of each subpopulation from the cohort. This classification model uses a combination of top-ranking analyte features, including proteins and lipids, with a total feature list of 43.

Given the limitations of CA125 alone, multi-omic models were explored to improve diagnostic performance by integrating lipidomics profiling with a panel of six protein biomarkers. In assessing feature space from the resulting 509 unique specimens, this multi-omic approach yielded a diverse range of lipid species and proteins that enabled modeling. Specifically, >100 gangliosides, >480 lipids and fatty acids, and 6 proteins were evaluated individually and in combination to identify the highest-performing models for OC classification using 20-fold cross-validation. Single biomarker classes alone resulted in AUCs ≤ 90% when comparing all non-OC groups (“All controls”) with all stages and subtypes of OC. In contrast, models incorporating all available analytes (“any” analyte) achieved a peak AUC of 95% (“All controls” vs. “OC”, Figure 1). These data indicate that combining lipids and proteins without feature class restriction enhances model performance. The final multi-omic model included fewer than 50 features including lipids, gangliosides, fatty acids, and proteins. Figure 1 illustrates performance across multiple subpopulations with average AUC and 95% confidence intervals (CIs). When comparing all controls to early-stage OC, the AUC remained very high at 94% (95% CI: 92–97%) in this difficult-to-detect population. Performance increased when analyzing only the “healthy” donors compared with OC (AUC of 98% with 95% CI: 97–100% for both early and all OC). Notably, performance in the population of benign adnexal masses and early-stage OC had an AUC of 92% (95% CI: 89–96%). The data represent a significant advancement in the early detection of OC in symptomatic women.

### 3.3. Assessing Preliminary Models and Clinical Performance

Clinical performance of the top-performing multi-omic models was evaluated across the cohort, focusing on fixed specificity levels of 70% and 80% to assess sensitivity. This approach reflects performance in real-world clinical scenarios, such as early detection or surgical triage, where balancing sensitivity and specificity is critical. Across both specificity thresholds, the model consistently achieved high sensitivity, exceeding 94% in most comparisons (Table 4).

When distinguishing all OC cases from all control groups, the model achieved 96.3% sensitivity at 70% specificity and 94.4% at 80% specificity. Sensitivity was even higher when comparing OC to healthy and gastrointestinal (GI) controls, reaching 98.6% at 70% specificity and 96.7% at 80% specificity. Performance remained robust when comparing OC to benign adnexal masses, achieving 94.4% at 70% specificity and 91.1% at 80% specificity. Importantly, early-stage OC was also detected with similarly high accuracy. Compared with all controls, early-stage sensitivity reached 96.1% and 94.8%; against healthy + GI controls, it reached 98.7% and 96.1%; and against benign controls, it reached 94.8% and 92.2% at 70% and 80% specificity, respectively. These results highlight the model’s capacity to detect early-stage disease while maintaining clinically acceptable specificity, even in the presence of diagnostically challenging benign adnexal masses.

### 3.4. Stage- and Treatment-Dependent Shifts in Biomarker Profiles

**Figure 2:**
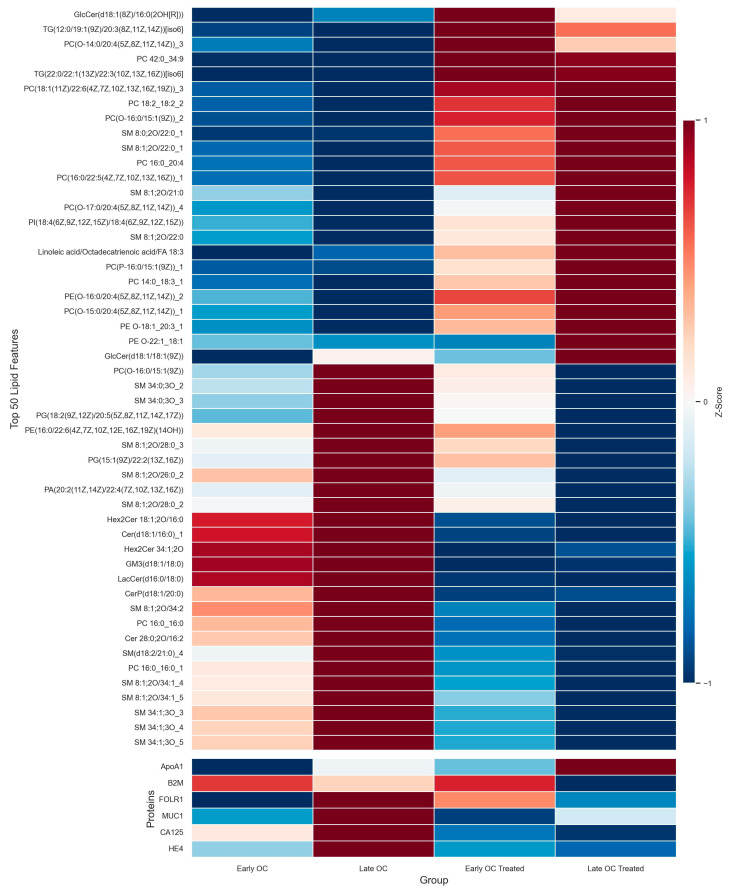
**Heatmap of top 50 ANOVA-selected lipid features and 6 ELISA-measured proteins across OC stage and treatment groups.** Lipid features were median normalized and auto-scaled (z-score normalization), while ELISA protein levels were auto-scaled prior to analysis. The top 50 features were selected based on one-way ANOVA across four groups: early-stage untreated (Early OC, *n* = 75), late-stage untreated (Late OC, *n* = 111), early-stage treated (Early OC Treat, *n* = 8), and late-stage treated (Late OC Treat, *n* = 24). Group-averaged abundance values are displayed in the heatmap. Hierarchical clustering using the Euclidean distance and Ward’s method shows that treated samples cluster together, as do untreated samples, indicating that treatment status is a major source of molecular variation across both lipid and protein markers.

Interestingly, the cohort contained a small subset of specimens obtained from early- and late-stage OC patients undergoing neoadjuvant chemotherapeutic treatment for their disease. This enabled an exploratory analysis where lipid and protein profile differences were assessed between newly diagnosed (“untreated”) and “treated” OC patients. Using one-way ANOVA, the top 50 lipid features that most significantly differed across early-stage untreated (Early OC, *n* = 75), late-stage untreated (Late OC, *n* = 111), early-stage treated (Early OC Treat, *n* = 8), and late-stage treated (Late OC Treat, *n* = 24) samples were identified. Group-averaged abundances of these lipid features, alongside six ELISA-measured proteins, were visualized in a heatmap (Figure 2).

The heatmap revealed that for lipids, early- and late-stage treated samples clustered together, forming a distinct group from the untreated early- and late-stage samples. However, protein abundance shifts in early-stage treated versus untreated samples were more variable and protein-specific, with some proteins increasing in abundance and others decreasing, resulting in less distinct clustering. In contrast, late-stage treated versus untreated samples exhibited more uniform and pronounced shifts across proteins, leading to clearer clustering separation.

Notably, proteins including CA125 and HE4, established clinical markers for OC monitoring, displayed similar treatment-associated shifts in abundance as the lipid features. The most striking observation was the late-stage treated group’s molecular profile, which exhibited a nearly inverse pattern relative to the late-stage untreated samples for both lipids and proteins alike. A comparable, but slightly less pronounced, pattern was also observed in early-stage OC, especially for lipids. These findings suggest that treatment induces substantial molecular changes. Although exploratory, it is hypothesized that this multi-omic platform, driven particularly by lipids for early-stage discrimination, could have clinical applications beyond earlier detection of OC in symptomatic women, such as monitoring therapeutic response.

## 4. Discussion

Ovarian cancer (OC) remains a significant clinical challenge, primarily due to its late-stage diagnosis, often resulting from a prolonged diagnostic odyssey in symptomatic women. Major population-based screening trials, such as the UK Collaborative Trial of Ovarian Cancer Screening (UKCTOCS) and the Prostate, Lung, Colorectal, and Ovarian (PLCO) Cancer Screening Trial, found no significant reduction in OC mortality from routine screening using CA125, transvaginal ultrasound (TVUS), or a multimodal strategy [28,29]. In UKCTOCS, the combination of CA125 with TVUS achieved a sensitivity of 89.5% and a specificity of 99.8%; however, this did not translate into a statistically significant mortality benefit [28]. As a result, current U.S. Preventive Services Task Force (USPSTF) and international guidelines recommend against population-wide screening for OC in asymptomatic women [30].

However, the lack of efficacy in population-wide screening should not be conflated with the potential utility of sensitive diagnostics in symptomatic patients [17]. In the United States, over three million women present with VAS annually, including bloating, early satiety, or pelvic discomfort, that overlap with early-stage OC but are often initially attributed to benign or gastrointestinal (GI) conditions [31]. These patients commonly face diagnostic delays of weeks to months, as providers pursue non-gynecologic workups or adopt a “wait-and-see” approach [9]. Deploying a high-sensitivity “rule out” assay at initial symptom presentation would enable rapid triaging and prioritizing of high-risk symptomatic individuals for imaging and specialist referral while avoiding unnecessary interventions in low-risk patients [32].

The results detailed here demonstrate that a multi-omic biomarker model combining lipids and proteins can accurately detect OC in a biologically complex, clinically relevant population. This cohort was deliberately enriched with diagnostically challenging cases, such as benign complex adnexal masses and advanced GI conditions. Lipids and proteins together enhanced detection of early-stage OC and were particularly effective in identifying HGSC, an aggressive subtype that can progress rapidly from undetectable precursor lesions to widespread disease within two years and is frequently missed by existing biomarkers [17,33,34]. The cohort included a substantial number of HGSC cases (Table S4), supporting the robustness of the model in this clinically critical subgroup. This multimodal approach, which improves detection across both stages and subtypes, offers a promising strategy for timelier OC diagnosis.

In contrast to DNA mutations or methylation-based tests under development for asymptomatic screening, the LC-MS-based approach described here is efficient, cost-effective, and fast. It requires minimal serum volume, uses only a single extraction reagent for sample preparation, and yields data from fewer than 50 features, facilitating rapid processing and simplified modeling by machine learning. The assay’s streamlined workflow and minimal sample requirements offer promise for implementation focused on low cost, speed, and accuracy.

By refining the diagnostic pathway for symptomatic women, this strategy addresses a core clinical gap and improves the chances of successful intervention. Modeling studies suggest that OC can progress from early lesions to disseminated disease in under two years, and annual screening may fail to intercept this rapid progression in time to influence outcomes [17,35,36]. However, timely detection in symptomatic women has been associated with improved survival and surgical outcomes. Importantly, the approach from this work aligns with emerging evidence supporting symptom-triggered testing. For example, a study by Kwong et al. showed that a fast-track pathway for symptomatic women led to early-stage (I/II) diagnoses in 25% of HGSC cases, a substantial improvement for a disease typically caught at late stages [37]. Notably, earlier diagnosis increases the likelihood of complete cytoreduction, a known predictor of survival, and ensures management by gynecologic oncologists, which has been shown to improve outcomes by as much as 12 months in median survival [38,39,40].

False positives generated through symptom-triggered triage are generally manageable and do not result in unnecessary surgeries [37,41,42]. Most are resolved through follow-up imaging and clinical evaluation, which are already part of the standard of care for adnexal mass assessment. TVUS, a low-risk, first-line imaging tool endorsed by major guidelines [43], serves as an ideal complement to biomarker-driven triage. In the model, false positives primarily occurred in patients with symptomatic adnexal masses, many of whom undergo surgery as part of standard care, suggesting minimal downstream clinical consequences. False negatives, by contrast, were distributed across both early- and late-stage cancers, indicating consistent model behavior but underscoring the need for ongoing optimization. As the targeted assay is developed, ongoing efforts are focused on refining feature selection, enhancing normalization through internal lipid standards, and calibrating decision thresholds tailored to specific clinical use cases.

To our knowledge, this is the first report of extensive lipid profiling in a symptomatic population of women; previous comparisons have focused on OC vs. healthy women, OC patient samples undergoing treatment and at various disease states for prognostic analyses, and OC vs. benign adnexal masses in women scheduled for surgery [44]. However, variability in pre-analytical factors that were not captured in this study, such as fasting state, body composition, or medication use, could not be assessed for lipid profiles. Nevertheless, these findings mark a significant step forward in the pursuit of earlier, more accurate diagnosis for OC. The multi-omic platform demonstrated 98.7% sensitivity in early-stage OC and 98.6% sensitivity across all subtypes at 70% specificity, representing a significant improvement over current detection tools. Such gains could have relevant clinical implications in not only improving patient outcomes but also reducing healthcare burden through earlier intervention and more targeted use of imaging and surgical resources.

In summary, these data support a shift toward precision-guided diagnostic triage in symptomatic women. This multi-omic serum assay offers a practical, scalable solution to a longstanding clinical problem, with potential to reduce diagnostic delay and enhance early-stage detection in women with signs and symptoms of OC. The exploratory analysis also revealed distinct molecular clustering between treated and untreated OC samples, particularly in late-stage disease. The observed molecular shifts highlight important biological changes induced by treatment, underscoring the need for further investigation in larger cohorts. A deeper understanding of how systemic therapies affect lipid and protein expression could refine biomarker panels and inform diagnostic strategies for OC across the clinical care continuum. Looking ahead, one promising extension of this platform could include clinical monitoring applications, particularly for lipid features [45]. As treatment landscapes evolve and biomarker panels and approaches improve performance, non-invasive, real-time molecular monitoring assays could be transformative in guiding surveillance and timely intervention, as well as early detection of disease.

## 5. Patents

Authors from AOA Dx disclose patent filings protecting claims of intellectual property of AOA Dx, Inc. within this manuscript.

## Figures and Tables

**Table 1:** **Tumor histology and cancer stage for cases and control diagnoses in the cohort (*N* = 509).** Ovarian cancer histologic subtypes and cancer stage are reported for cases (*n* = 215). Clinical control diagnoses are shown (*n* = 294) and include benign gynecologic conditions, GI disorders, and samples from individuals with no reported abnormalities.

	*N* = 509			
	Cases		Controls	
	*n* = 215		*n* = 294	
	*n*	%	*n*	%
Histology				
High-Grade Serous	141	65.9		
Low-Grade Serous	10	4.7		
Unspecified	17	7.9		
Endometrioid	16	7.5		
Mucinous	13	6.1		
Clear Cell	11	5.1		
Mixed	5	2.3		
Non-Epithelial	1	0.5		
Benign			165	56.1
Normal			80	27.2
GI Disorder			49	16.7
Stage—All OC				
I	52	24.3		
II	25	11.7		
III	120	56.1		
IV	16	7.5		
Unstaged	1	0.5		

**Table 2:** **Demographic characteristics of clinically annotated serum samples in the cohort (*N* = 509).** Age distribution and race/ethnicity are shown for ovarian cancer cases (*n* = 215) and clinical controls (*n* = 294). The cohort includes both pre- and postmenopausal women. For context, the average age at ovarian cancer diagnosis is 63, and risk increases with age [24,25].

	*N* = 509			
	Cases		Controls	
	*n* = 215		*n* = 294	
	*n*	%	*n*	%
Age				
<50	39	18.2	119	40.5
50–59	71	33.2	96	32.7
60–69	66	30.8	50	17.0
70+	37	17.3	28	9.5
Unknown	1	0.5	0	0.0
Mean (sd)				
Race/Ethnicity				
Non-Hispanic	117	54.7	16	5.4
Caucasian	69	32.2	228	77.6
Hispanic	25	11.7	2	0.7
Unknown	2	0.9	46	15.6
Asian	1	0.5	0	0.0
African-American	0	0.0	2	0.7

**Table 3:** **CA125 performance across multiple decision points.** Data are presented according to current guidelines with a cut-off at 35 U/mL. Menopausal status is categorized by age: >50 years = postmenopausal; ≤50 years = premenopausal.

Group Comparison	All Ages		>50 Years Old		≤50 Years Old	
	Sensitivity	Specificity	Sensitivity	Specificity	Sensitivity	Specificity
**All Controls vs.** **All OC**	76.2%	90.1%	77.4%	92.1%	72.0%	87.6%
**Benign Adnexal Mass vs. All OC**	76.2%	82.7%	77.4%	86.0%	72.0%	66.7%
**All Controls vs.** **Early-Stage OC**	67.5%	90.1%	69.4%	92.1%	60.0%	87.6%

**Table 4:** **Sensitivity and specificity for multi-omics modeling**. Multiple comparisons of interest were evaluated to determine sensitivity (SN) at 70% and 80% specificity (SP) targets.

Comparison	SN (%) at 70% SP	SN (%) at 80% SP
**All Controls vs. All OC**	96.3%	94.4%
**Healthy + GI vs. All OC**	98.6%	96.7%
**Benign vs. All OC**	94.4%	91.1%
**All Controls vs. Early-Stage OC**	96.1%	94.8%
**Healthy + GI vs. Early-Stage OC**	98.7%	96.1%
**Benign vs. Early-Stage OC**	94.8%	92.2%

## Data Availability

The data supporting the conclusions of this article may be made available by the authors on request.

## Supplementary Materials

The following supporting information can be downloaded at https://www.mdpi.com/article/10.3390/diagnostics15172225/s1; Table S1. Distribution of benign gynecologic and adnexal conditions in the study cohort (N = 165). The most frequently observed diagnoses included fibroma (13.33%), cysts (11.52%), and endometrioid cysts (10.3%). The table encompasses a range of benign neoplasms, cystic lesions, hyperplastic conditions, and findings associated with hereditary cancer syndromes. Table S2. Gastrointestinal (GI) conditions in the study cohort (N = 49). Ulcerative colitis and Crohn’s disease were the most prevalent diagnoses, each accounting for 42.9% of cases. Less common conditions included diverticulitis with complications, chronic inflammation, and other GI pathologies. Table S3. Age distribution by clinical group within the cohort (N = 509). Groups include benign conditions, late- and early-stage OC, healthy controls, and gastrointestinal (GI) disorders. Age is presented as counts by category (<50, 50–59, 60–69, 70+), along with the number of unknown entries, mean age, standard deviation (StDev), and total participants per group. Table S4. Distribution of serous ovarian cancer cases by histological stage. High-grade and low-grade serous ovarian cancer cases (n = 151) are stratified by pathological stage (I–IV and unstaged) within the cohort. This breakdown highlights the predominance of late-stage presentation in high-grade serous cases, consistent with established clinical patterns.

## Abbreviations

The following abbreviations are used in this manuscript:

ApoA1	Apolipoprotein A1
B2M	Beta-2 microglobulin
CA125	Cancer Antigen 125
CI	Confidence interval
CV	Coefficient of variation
ESI	Electrospray ionization
FOLR1	Folate receptor alpha
GI	Gastrointestinal
GU	Genitourinary
HE4	Human epididymis protein 4
HGSC	High-Grade Serous Carcinoma
LC-MS	Liquid chromatography–mass spectrometry
ML	Machine Learning
MUC1	Mucin 1
OC	Ovarian Cancer
PLCO	Prostate, Lung, Colorectal, and Ovarian Cancer Screening Trial
SN	Sensitivity
SP	Specificity
TVUS	Transvaginal ultrasound
UKCTOCS	UK Collaborative Trial of Ovarian Cancer Screening
USPSTF	U.S. Preventive Services Task Force
VAS	Vague Abdominal Symptoms
