# Supplementary material for: Clinical Evaluation of a Multi-Omic Diagnostic Model for Early-Stage Ovarian Cancer Detection
Source: Diagnostics (Basel). 2025 Sep 2;15(17):2225. doi: 10.3390/diagnostics15172225 (PMC12428613; doi:10.3390/diagnostics15172225)
Supplement: Supplementary file 1 [file diagnostics-15-02225-s001.zip › diagnostics-3755334-supplementary.pdf]

Table S1. Distribution of benign gynecologic and adnexal conditions in the study cohort (N = 165). The most frequently observed diagnoses included fibroma (13.33%), cysts (11.52%), and endometrioid cysts (10.3%). The table encompasses a range of benign neoplasms, cystic lesions, hyperplastic conditions, and findings associated with hereditary cancer syndromes.

Benign conditions	Count	%
fibroma	22	13.3
cyst	19	11.5
endometrioid cyst	17	10.3
Serous cystadenoma	12	7.3
mature teratoma	11	6.7
serous cyst	10	6.0
benign adnexal mass	8	4.8
atypical hyperplasia	7	4.2
follicular cyst	6	3.6
serous cystadenofibroma	5	3.0
Leiomyoma	4	2.4
dermoid cyst	4	2.4
mucinous cystadenoma	3	1.8
benign	3	1.8
serous papillary cystadenoma	3	1.8
endometrioid cystadenoma	3	1.8
benign—therapeutic bilateral salpingo-oophorectomy, breast CA BRCA2 positive	2	1.2
serous cysts	1	0.6
adenomyosis, leiomyoma	2	1.2
tecofibroma	2	1.2
thecoma	2	1.2
fibrothecoma	2	1.2
endometrial polyp	2	1.2
endometrial hyperplasia, adenomyosis	1	0.6
cystadenoma	1	0.6
benign—therapeutic bilateral salpingo-oophorectomy, breast CA ER/PR positive	1	0.6
benign—therapeutic bilateral salpingo-oophorectomy, breast CA BRCA1 positive	1	0.6
benign—BRCA2 positive	1	0.6
typical hyperplasia, endometrial polyp	1	0.6
benign—history of stage IIA ovarian adenocarcinoma	1	0.6
hyperplasia	1	0.6
Adenofibroma	1	0.6
adenomyosis	1	0.6
chronic endometritis	1	0.6
typical hyperplasia, adenomyosis	1	0.6
benign—history of breast cancer, BRCA2 positive	1	0.6
epithelial metaplasia	1	0.6
cystadenofibroma	1	0.6
**Grand Total**	**165**	**100.0**

Table S2. Gastrointestinal (GI) conditions in the study cohort (N = 49). Ulcerative colitis and Crohn's disease were the most prevalent diagnoses, each accounting for 42.9% of cases. Less common conditions included diverticulitis with complications, chronic inflammation, and other GI pathologies.

**GI Conditions**	**Count**	**%**
Ulcerative colitis	21	42.9
Crohn's disease	21	42.9
Diverticulitis	2	4.1
Ruptured sigmoid diverticulitis with abscess formation and dense fibrous adhesions	1	2.0
Diverticulitis with pericolonic abscess formation and adhesions, involving the fallopian tube and ovary	1	2.0
Acute and chronic inflammation	1	2.0
Crohn's colitis, negative for dysplasia	1	2.0
Active chronic gastritis	1	2.0
**Grand Total**	**49**	**100.0**

Table S3. Age distribution by clinical group within the cohort (N = 509). Groups include benign conditions, late- and early-stage OC, healthy controls, and gastrointestinal (GI) disorders. Age is presented as counts by category (<50, 50–59, 60–69, 70+), along with the number of unknown entries, mean age, standard deviation (StDev), and total participants per group.

**Age Distribution by Group**									
**Group**	**<50**	**50–59**	**60–69**	**70+**	**Unknown**	**Mean**	**StDev**	**Total**	**%**
Benign	70	56	24	15	0	33	29.1	165	32.4%
Late-Stage OC	26	43	41	24	0	26.8	17.3	134	26.3%
Early-Stage OC	13	28	25	14	1	16.2	10.8	81	15.9%
Healthy	25	36	17	2	0	16	15.3	80	15.7%
GI Disorder	25	4	9	4	7	9.8	8.8	49	9.6%
Total	159	167	116	59	8	101.8	67.7	509	100.0%

Table S4. Distribution of serous ovarian cancer cases by histological stage. High-grade and low-grade serous ovarian cancer cases (n = 151) are stratified by pathological stage (I–IV and unstaged) within the cohort. This breakdown highlights the predominance of late-stage presentation in high-grade serous cases, consistent with established clinical patterns.

	**Pathological Stage**					
**Serous Ovarian Cancer**	**I**	**II**	**III**	**IV**	**Unstaged**	**Total**
High-Grade Serous	17	15	95	13	1	141
Low-Grade Serous	1	1	7	1		10
